# Gene expression signature of cerebellar hypoplasia in a mouse model of Down syndrome during postnatal development

**DOI:** 10.1186/1471-2164-10-138

**Published:** 2009-03-30

**Authors:** Julien Laffaire, Isabelle Rivals, Luce Dauphinot, Fabien Pasteau, Rosine Wehrle, Benoit Larrat, Tania Vitalis, Randal X Moldrich, Jean Rossier, Ralph Sinkus, Yann Herault, Isabelle Dusart, Marie-Claude Potier

**Affiliations:** 1 Laboratoire de Neurobiologie, CNRS UMR7637, ESPCI, Paris, France; 2 Equipe de Statistique Appliquee – ESPCI, Paris, France; 3 Neurobiologie des Processus Adaptatifs, CNRS UMR7102, Paris, France; 4 UPMC, Paris, France; 5 Laboratoire Ondes et Accoustique, UMR7587, ESPCI, Paris, France; 6 The Queensland Brain Institute, St Lucia, Australia; 7 IEM, CNRS UMR6218, Orleans, France; 8 CRICM, CNRS UMR7225, INSERM UMR975, UPMC, CHU Pitie-Salpetriere, Paris, France

## Abstract

**Background:**

Down syndrome is a chromosomal disorder caused by the presence of three copies of chromosome 21. The mechanisms by which this aneuploidy produces the complex and variable phenotype observed in people with Down syndrome are still under discussion. Recent studies have demonstrated an increased transcript level of the three-copy genes with some dosage compensation or amplification for a subset of them. The impact of this gene dosage effect on the whole transcriptome is still debated and longitudinal studies assessing the variability among samples, tissues and developmental stages are needed.

**Results:**

We thus designed a large scale gene expression study in mice (the Ts1Cje Down syndrome mouse model) in which we could measure the effects of trisomy 21 on a large number of samples (74 in total) in a tissue that is affected in Down syndrome (the cerebellum) and where we could quantify the defect during postnatal development in order to correlate gene expression changes to the phenotype observed. Statistical analysis of microarray data revealed a major gene dosage effect: for the three-copy genes as well as for a 2 Mb segment from mouse chromosome 12 that we show for the first time as being deleted in the Ts1Cje mice. This gene dosage effect impacts moderately on the expression of euploid genes (2.4 to 7.5% differentially expressed). Only 13 genes were significantly dysregulated in Ts1Cje mice at all four postnatal development stages studied from birth to 10 days after birth, and among them are 6 three-copy genes. The decrease in granule cell proliferation demonstrated in newborn Ts1Cje cerebellum was correlated with a major gene dosage effect on the transcriptome in dissected cerebellar external granule cell layer.

**Conclusion:**

High throughput gene expression analysis in the cerebellum of a large number of samples of Ts1Cje and euploid mice has revealed a prevailing gene dosage effect on triplicated genes. Moreover using an enriched cell population that is thought responsible for the cerebellar hypoplasia in Down syndrome, a global destabilization of gene expression was not detected. Altogether these results strongly suggest that the three-copy genes are directly responsible for the phenotype present in cerebellum. We provide here a short list of candidate genes.

## Background

Down syndrome (DS) results from the presence in three copies of human chromosome 21, the smallest human autosome containing about 350 known protein-coding genes [1-4]. The mechanisms by which this aneuploidy produces the complex and variable phenotype observed in DS patients are still under discussion.

The use of large scale gene expression methods such as microarrays were expected to shed light on which genes (within or outside chromosome 21) contribute to the DS phenotype as well as to the phenotypic variability.

For the genes on chromosome 21, all studies have confirmed a general increase of transcription following the chromosomal imbalance, or "primary gene dosage effect". RNA samples prepared from cells or tissues of DS patients or mouse models showed a global over-expression of the three-copy genes [5-15]. However, even if the mean over-expression was generally reported to be close to the expected value of 1.5, recent studies in DS cell lines have reported that about 70% of the three-copy genes were significantly below the 1.5 ratio. In these particular cell lines at least, a large proportion of the chromosome 21 transcripts were compensated for the primary gene dosage effect [6,16].

As for non-chromosome 21 genes, results are less consistent. The aneuploidy of an entire chromosome could affect the expression of either a limited number genes or a large number in a more random and extensive way [9,13]. This question is still debated and more comprehensive studies assessing the variability among samples, tissues and development stages are needed.

We thus designed a large scale gene expression study in which we could measure the effects of trisomy 21 on a large number of samples in a tissue that is affected in DS where we could quantify the defect during postnatal development in order to correlate gene expression changes with the phenotype observed.

The Ts1Cje mouse model of DS is a segmental trisomy of mouse chromosome 16 with many genes orthologous to human chromosome 21 present in three copies (about 95). This mouse model has the advantage of being available as large colonies of mice on B6C3SnF1/Orl mixed genetic background and rapidly screened [17]. We focused our study on cerebellum since adult Ts1Cje mice show a reduction in cerebellar volume that parallel the observations in DS patients and in another mouse model of DS (Ts65Dn mice) [18,19]. The reduced size of the cerebellum and the reduced cerebellar granule cell number in Ts65Dn adults originate around birth because of a defect in granule cell precursor proliferation [20]. In the present study four early postnatal time points that are crucial for cerebellar development were investigated which could provide a read-out of genes involved in cerebellar hypoplasia in DS. These four time points correspond to birth (P0) and postnatal days 3 (P3), 7 (P7) and 10 (P10). During this time period granule cells proliferate and migrate from the external to the internal granule cell layer and Purkinje cells start differentiating and growing their highly dense dendritic tree. We quantified the proliferation of granule cell precursors on fixed cerebellum slices of Ts1Cje and euploid mice at P0, P3 and P7 using immunohistochemistry and histology. A significant 30% decrease of their mitotic index was observed at P0 but not at P3 and P7, in agreement with the results obtained in Ts65Dn mice [20]. Finally and in order to find gene expression variations in granule cell precursor rich cerebellar regions, external granule cell layers of newborn Ts1Cje and euploid mice were dissected and analyzed on microarrays.

## Results

### The cerebellar volume in Ts1Cje adult mice is reduced

Using magnetic resonance imaging (MRI) we measured the volumes of whole brain and cerebellum of six male adult Ts1Cje and nine euploid littermates with a very high-resolution of 100 *μ*m (see Additional file 1). Total brain volumes were not different between trisomic and euploid mice (Table 1). However we found a significant 16% decrease in the cerebellum volume of Ts1Cje mice as compared to euploids (*p *= 0.028; two-sided *t*-test).

**Table 1:** Cerebellar size in adult Ts1Cje and euploid mice.

	Brain	Cb	normalized Cb
Euploid (n = 9)	599 ± 24.2	68 ± 7.1	0.114 ± 0.013
Ts1Cje (n = 6)	608 ± 20.4	57 ± 7.3	0.094 ± 0.014
Volume ratio	102%	84%	83%
*p*-value (two sided *t*-test)	0.47	0.028	0.028

Average volumes are given in cubic mm; Cb = cerebellum; brain volume includes Cb. Normalized Cb refers to the Cb volume divided by the total brain volume.

### Granule cell proliferation is decreased in newborn Ts1Cje mice

**Figure 1:**
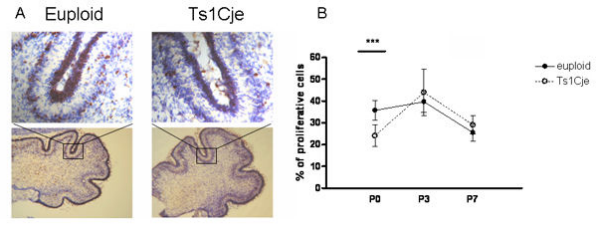
**Proliferation of granule cells in the cerebellum of Ts1Cje and euploid mice during postnatal development**. (A) Ki-67 immunoreactivity (brown) over Cresyl violet coloration (blue) in P0 cerebellar sections from Ts1Cje and euploid mice. (B)Index of proliferation in the external granule cell layer of the cerebellum shows a significant 32.6% decrease in Ts1Cje mice as compared to euploids at P0 (***p < 0.001; *t*-test). Index of proliferation corresponds to the number of Ki67 positive cells divided by the total number of cells as determined using Cresyl violet counterstaining. n = 4 for each condition.

Granule cell proliferation was measured in the cerebellum of Ts1Cje and euploid mice at P0, P3 and P7 at a time when granule cell precursors proliferate in the external granule cell layer. Cryostat sections of cerebellar cortex were immunostained using an antibody against Ki67 (in brown on Figure 1A), a specific cellular proliferation marker [21]. Mitotic index was calculated as the number of Ki67-positive cells divided by the total cell number determined by Cresyl violet counterstaining. We observed a significant 32.6% reduction of the mitotic index in the external granule cell layer of Ts1Cje mice at P0 (*p *< 0.001; two-sided *t*-test) but not at P3 and P7 (Figure 1B). This result is similar to the 22% decrease found in the cerebellum of Ts65Dn mice at birth but not at P6 using hematoxylin and cresyl violet stainings [20]. Altogether these data suggest a similar early event occurring around birth that affects granule cell proliferation in the cerebellum of Ts1Cje and Ts65Dn mice. We did not observe any change in the number of pyknotic nuclei between Ts1Cje and euploid mice (data not shown).

### Gene expression changes in the cerebellum of Ts1Cje and wild type mice between P0 and P10

In order to find genes or pathways involved in the cerebellar hypoplasia, we analyzed gene expression in the cerebellum of Ts1Cje and euploid mice at P0, P3, P7 and P10 using pangenomic two-color microarrays containing 25 344 probes representing approximately 15 574 mouse genes. The main objective was to detect differentially expressed genes between Ts1Cje and euploid samples. Thus we hybridized Ts1Cje versus euploid samples at each postnatal developmental stage (four experiments at each time point using a total of 16 Ts1Cje and 16 euploid mice). In order to estimate the impact of postnatal development on gene expression while still evaluating the difference between Ts1Cje and euploid samples with a minimum of experiments, we performed an additional series of comparisons between Ts1Cje and euploid samples at two successive stages (four experiments P0 versus P3, P3 versus P7 and P7 versus P10 using a total of 12 Ts1Cje and 12 euploid mice) (see Additional file 2). Fifty-six samples from individual cerebella were hybridized on 28 microarrays. To detect genes differentially expressed during cerebellar postnatal development and/or in Ts1Cje mice, we performed an analysis of variance (ANOVA) with two factors: the postnatal developmental stage (P0, P3, P7 or P10) and the genotype (Ts1Cje or euploid)(Table 2). In addition we compared the expression of 22 previously selected genes in Ts1cje and euploid samples at P0 using quantitative real-time PCR (qPCR).

**Table 2:** ANOVA parameters of gene expression levels.

	P0	P3	P7	P10
Euploid	*μ*	*μ *+ *α*3	*μ *+ *α*7	*μ *+ *α*10
Ts1Cje	*μ *+ *β*0	*μ *+ *α*3 + *β*3	*μ *+ *α*7 + *β*7	*μ *+ *α*10 + *β*10

Our model considers the effects of 2 factors: the development (*α*_*i *_parameters) and the trisomy effect (*β*_*i *_parameters). *μ *represent the mean gene expression.

#### Effect of trisomy on gene expression at P0, P3, P7 and P10

In Ts1Cje mice at P0, P3, P7 and P10, between 372 and 1164 genes were found to be significantly differentially expressed (*p *< 0.05; summarized in Table 3; see Additional file 3). The differentially expressed genes represented between 2.4% to 7.5% of the expressed genes present on the microarray. These results indicate that trisomy does not lead to a significant global change in gene expression during early cerebellar postnatal development.

**Table 3:** Number of differentially expressed genes in the postnatal cerebellum of Ts1Cje mice.

	Trisomy effect				Developmental effect		
	P0	P3	P7	P10	P0 → P3	P0 → P7	P0 → P10
Expressed genes	15458	15552	15574	15463	15368	15565	14208
2 N differentially expressed genes	824	1164	372	532	1476	1930	1592
3 N differentially expressed genes	11	13	12	11	4	3	2

Number of differentially expressed genes in Ts1Cje mice at P0, P3, P7 and P10 (Trisomy effect) and during normal cerebelelar development P0–P3, P0–P7 and P0–P10 (Developmental effect) obtained by ANOVA(*α *= 5%).

We then listed genes that were dysregulated in Ts1Cje cerebella at all four postnatal development stages studied and found only 13, with 12 up-regulated genes (Table 4 and see Additional file 4). Among them, 6 are triplicated genes. One of them, Son or 'Son cell proliferation protein', had been previously reported as a candidate gene for DS cerebellar phenotype [8].

**Table 4:** List of genes differentially expressed at P0, P3, P7 and P10 in the cerebellum of Ts1Cje mice as compared to euploids.

Symbol	P0	P3	P7	P10	Name	Gene Ontology
*1700021N20Rik*	0.79	1.43	0.71	0.75	RIKEN cDNA 1700021N20	
***Atp5o***	**1.27**	**1.26**	**1.59**	**1.46**	**ATP synthase, H+ trans- porting, mitochondrial F1 complex, O subunit**	**hydrogen-transporting ATP synthase activity; mitochondrion; catalytic core F(1); ion transport; metal ion binding**
*Bpil3*	1.19	1.24	1.25	1.23	bactericidal/permeability- increasing protein-like 3	lipid binding
*Dnahc11*	2.42	2.63	3.94	3.02	dynein, axonemal, heavy chain 11	axonemal dynein complex; cilium;determination of left/right symmetry; microtubule motor activity
*Hspy2l*	1.40	1.29	1.44	1.46		protein folding; DNA bind ing;transcription; nucleus
***Ifngr2***	**1.26**	**1.51**	**1.28**	**1.47**	**interferon gamma receptor 2**	**receptor activity**
***Girk2***	**1.21**	**1.40**	**1.31**	**1.30**	**potassium inwardly- rectifying channel, sub- family J, member 6**	**G-protein activated inward rectifier potassium channel activity; integral to plasma membrane; potassium ion transport; voltage-gated ion channel activity**
*March1*	1.40	1.23	1.36	1.37	membrane-associated ring finger (C3HC4) 1	ubiquitin cycle; zinc ion binding
***Pcp4***	**1.50**	**1.41**	**1.70**	**1.53**	**Purkinje cell protein 4**	**calcium ion binding**
*Phtf1*	1.60	1.26	1.40	1.37	putative homeodomain transcription factor 1	DNA binding, transcription factor activity; nucleus
***Sod1***	**1.56**	**1.43**	**1.89**	**1.64**	**superoxide dismutase 1, soluble**	**removal of superoxide radicals;response to oxidative stress; copper, zinc superoxide dismutase activity;mitochondrion; DNA fragmentation during apoptosis; activation of MAPK activity**
***Son***	**1.32**	**1.48**	**1.60**	**1.53**	**Son cell proliferation pro tein**	**protein binding; intracellular; DNA binding;double-stranded RNA binding; nucleus**
*V1ri10*	1.37	1.30	1.56	1.46	vomeronasal 1 receptor, I10	pheromone receptor activity; integral to plasma membrane

All ratios have a *p*-value < 0.05 (ANOVA). In bold are three-copy genes.

#### Effect of development (10 days postnatally) on gene expression

Between P0 and P3, P0 and P7 and P0 and P10, the following number of genes were differentially expressed, respectively, 1476, 1930 and 1592, representing about 9.6 to 12.4% of expressed genes (Table 3). These results demonstrate that postnatal development impacts on the expression of a greater number of genes than trisomy does, as already seen in previous analysis [10].

#### Combined effects of trisomy and postnatal development

A high proportion of genes whose expression is regulated during cerebellar postnatal development were also differentially expressed in Ts1Cje mice at least at one time point (1187 genes). Among these, 3 are triplicated genes: *Girk2*, *Olig1 *and *Dscam *(Table 5). *Girk2 *is the only triplicated gene differentially expressed during cerebellar postnatal development and constantly over-expressed in Ts1Cje mice between P0 and P10. *Girk2*, also know as *Kcnj6*, is a voltage-gated potassium channel mainly expressed in granule cells of the cerebellum [22]. A point mutation of *Girk2 *in Weaver mice (wv) leads to granule cell degeneration [23]. *Olig1 *is a transcription factor involved in neuron fate commitment [24] and *Dscam *is a cell-surface receptor implicated in cell adhesion [25].

**Table 5:** List of three-copy genes overexpressed in Ts1Cje mice and differentially expressed during cerebellar development.

	Trisomy effect				Developmental effect		
	P0	P3	P7	P10	P0 → P3	P0 → P7	P0 → P10
Girk2	1.21	1.40	1.31	1.29	1.50	3.31	3.11
Olig1	1.47	ns	1.34	ns	1.88	ns	ns
Dscam	ns	1.41	ns	ns	0.76	ns	ns

Expression ratio between trisomic and euploid mice (trisomy effect) and betwen two time points (development effect). (ANOVA, *α *= 5%). NS: not significant.

#### Effect of trisomy on the expression of genes involved in neuron proliferation

**Figure 2:**
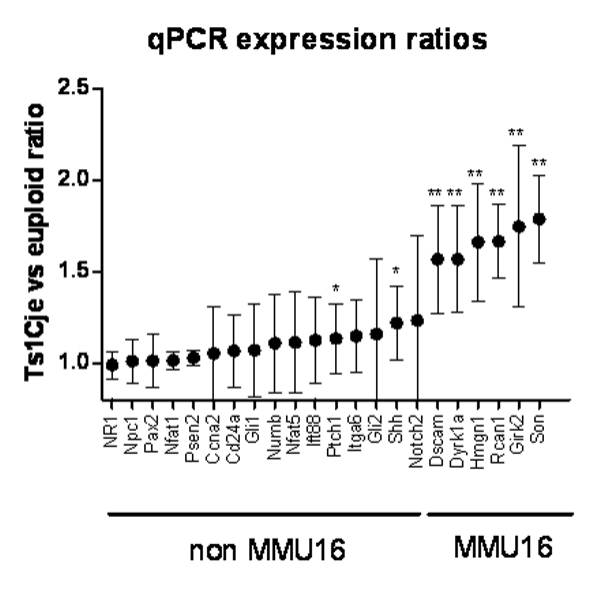
**qPCR gene expression ratio of candidate genes between Ts1Cje and euploid cerebella at P0**. Difference in expression of 22 genes, including 6 from Mmu16, between cerebella of 6 Ts1Cje and 6 euploid mice at P0 using real-time qPCR. Genes are ordered on the x axis according to their expression ratio. (*p < 0.05 and **p < 0.01; *t*-test).

In order to identify specific pathways involved in cerebellar postnatal development and disrupted in Ts1Cje mice, we performed qPCR experiments on a selection of genes using the cerebellum of 6 Ts1Cje mice and 6 euploid mice from three litters at birth. The list contained 22 genes belonging to pathways involved in cell proliferation and differentiation of the cerebellum or directly in the DS cerebellar phenotype [26]: the Notch signalling pathway [27], *Numb *[28], *Gli1*, *Gli2 *and *Ptch1 *involved in the Shh signaling pathway [29], *Nfat1 *and *Nfat5 *[30] and *Rcan1 *[31] regulating Ca^2+ ^signalling and *Son *and *Hmgn1*, two triplicated genes that have been proposed to be candidate genes for the cerebellar phenotype [8]. Expression ratios between Ts1Cje and euploid cerebellum are shown in Figure 2. The six triplicated genes tested were significantly over-expressed with a mean ratio greater than 1.5. Surprisingly, of the 16 euploid genes tested, 2 were found to be slightly but significantly over-expressed: *Shh *(mean ratio = 1.16) and its receptor *Ptch1 *(mean ratio = 1.08).

### Major primary effect of trisomy exists in the external granule cell layer

**Figure 3:**
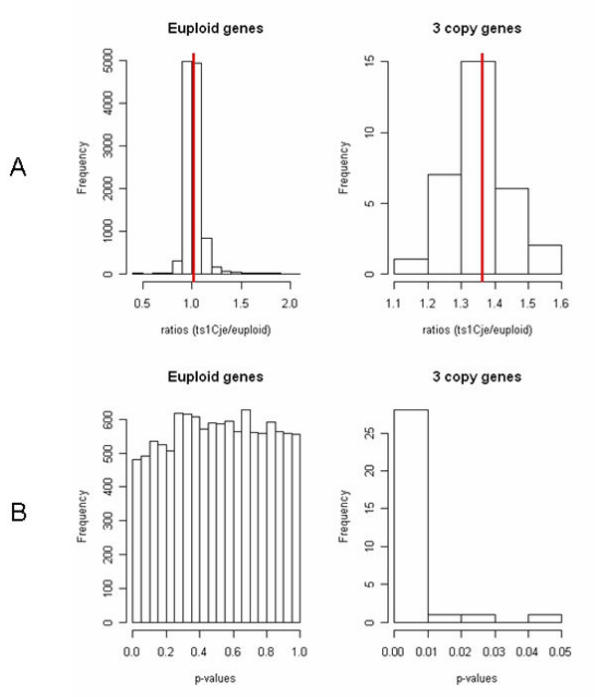
**Distribution of gene expression ratios (Ts1Cje/euploid) and their p-values in the external granule cell layer at P0**. Distributions of ratios (A) and their corresponding *p*-values (B) for all the genes analyzed (11 305) and for the three-copy genes only (41); *p*-values refer to the *t*-test.

**Figure 4:**
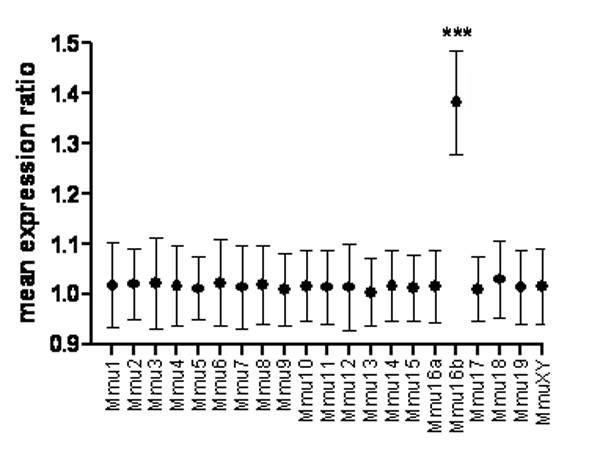
**Mean gene expression ratio according to chromosomal location**. Mean expression ratio (Ts1Cje/euploid) was significant only with Mmu16b genes (mean = 1.38, *p *< 0.001, *t*-test). Mmu16a and Mmu16b refer to euploid and three-copy genes in the Ts1Cje mice respectively.

**Figure 5:**
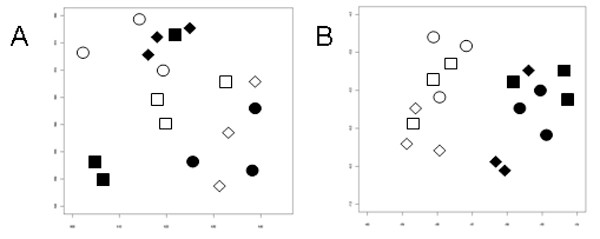
**PCA of gene expression data from the external granule cell layer of Ts1Cje and euploid mice at P0**. Principal Component Analysis on 11 305 genes expressed in the external granule cell layer at P0 (A) and on the 41 three-copy genes (B). Open and filled symbols correspond to euploid and Ts1Cje mice respectively. Circles, squares and lozenges refer to the three litters used.

In order to find gene expression variations in specific regions of the cerebellum where proliferation is impaired in Ts1Cje mice, external granule cell layers of 9 newborn Ts1Cje and 9 euploid mice from three litters were dissected and analyzed individually on microarrays. We used Illumina pangenomic microarrays containing more than 45 200 transcripts corresponding to about 19 100 genes. Gene expression data were analyzed using *t*-test (*α *= 5%). Of the 11 305 expressed genes only 4% (479 genes) were found to be significantly differentially expressed, with ratios between 0.44 and 2.06. This low percentage was similar to those observed in the entire cerebellum at P0, P3, P7 and P10. As shown on Figure 3A, the distribution of expression ratios for euploid genes was approximately 1.0 (1.02 ± 0.08) while the distribution of the three-copy genes was shifted towards higher values corresponding to the gene dosage effect (1.38 ± 0.11). Figure 4 shows the mean expression ratios for each individual chromosome. Only the part of Mmu16 present in three copies in Ts1Cje (noted as MMU16b) gave a mean expression ratio that was significantly different from 1.0 (*p *< 0.001; *t*-test). No difference was observed in the expression ratio variances, particularly between the MMU16b and all other chromosomes. The distributions of *p*-values were also very different for the euploid and the three-copy genes. For the euploid genes the *p*-value distribution was uniform indicating that very few genes were differentially expressed. However for the three-copy genes, all *p*-values were below 0.05 with a higher proportion under 0.01. All three-copy genes expressed in the external granule cell layer at P0 were found to be significantly over-expressed (*t*-test, *α *= 5%) (Figure 3, Table 6, Table 7). In order to reduce the number of false positives that occur during multiple testing, we applied the False Discovery Rate (FDR) controlling procedure of Benjamini and Hochberg on the *t*-test *p*-values [32]. As reported in Table 6, we found only 33 significantly dysregulated genes (28 over-expressed and 5 repressed). Of these 32 genes, 23 are three-copy genes, representing more than 70% of the dysregulated genes. The other nine are located on eight other chromosomes and are related to ion transport, signal transduction, cell proliferation or DNA processes. Altogether these results show a major primary gene dosage effect on the three-copy genes. This effect was also clearly detected using Principal Component Analysis (PCA). PCA is a technique used to represent multidimensional data sets along principal component axes that reffect the degree of variance in the data. Ts1Cje and euploid samples were grouped separately only when considering the three-copy genes (Figure 5).

**Table 6:** List of genes differentially expressed in the external granule cell layer of Ts1Cje mice at P0 (*q *< 0.05)

SYMBOL	Ratio	*p*-value	*q*-value	Chr	GO biological process category
***1810007M14Rik***	**1.53**	**0,000**	**0,004**	**16**	**Regulation of transcription**
*2400009B08Rik*	0.8	0	0,029	8	
***2610039C10Rik***	**1.5**	**0,000**	**0,038**	**16**	
***Atp5o***	**1.27**	**0,000**	**0,001**	**16**	**ATP biosynthetic process, H+ transport**
***B3galt5***	**1.4**	**0,000**	**0,014**	**16**	**Protein amino acid glycosylation**
***Brwd1***	**1.35**	**0,000**	**0,036**	**16**	**Regulation of transcription**
***Cbr1***	**1.32**	**0,000**	**0,007**	**16**	**Carbonyl reductase (NADPH) activity**
*Cdca7l*	0.46	0	0	12	Cell proliferation, transcription
***Chaf1b***	**1.43**	**0,000**	**0,036**	**16**	**Cell cycle, DNA replication, Transcrip tion**
***Dscam***	**1.47**	**0,000**	**0,029**	**16**	**Cell adhesion, Nervous system develop ment**
***Dyrk1a***	**1.35**	**0,000**	**0,003**	**16**	**Peptidyl-tyrosine phosphorylation**
*Entpd3*	0.84	0	0,028	9	Nucleoside diphosphate catabolic process
***Ets2***	**1.49**	**0,000**	**0,021**	**16**	**Regulation of cell cycle**
*Gin1*	1.19	0	0,007	1	DNA recombination
*Gng4*	0.63	0	0,002	13	Signal transduction
*Hfm1*	1.29	0	0,016	5	
***Hlcs***	**1.33**	**0,000**	**0,003**	**16**	**Protein modification process**
***Ifngr2***	**1.35**	**0,000**	**0,001**	**16**	
***Itsn1***	**1.26**	**0,000**	**0,003**	**16**	**Regulation of Rho protein signal transduction Endocytosis**
*Kcns2*	1.19	0	0,044	15	
***Mrps6***	**1.43**	**0,000**	**0,001**	**16**	**Translation**
*OTTMUSG00000018617*	1.65	0	0,001	19	
***Pigp***	**1.33**	**0,000**	**0,022**	**16**	**GPI anchor biosynthetic process**
***Psmg1***	**1.43**	**0,000**	**0,003**	**16**	**Cell proliferation**
***Rcan1***	**1.37**	**0,000**	**0,003**	**16**	**Ca2+ mediated signaling**
***Setd4***	**1.25**	**0,000**	**0,011**	**16**	
***Sfrs15***	**1.39**	**0,000**	**0,029**	**16**	
***Sod1***	**1.59**	**0,000**	**0,001**	**16**	**Negative regulation of neuron apoptosis Response to oxidative stress**
*Sp4*	0.62	0	0,001	12	
***Tmem50b***	**1.42**	**0,000**	**0,001**	**16**	
***Ttc3***	**1.29**	**0,000**	**0,036**	**16**	**zinc ion binding**
***Wrb***	**1.4**	**0,000**	**0,001**	**16**	

32 genes were found significantly differentially expressed by *t*-test (*p *< 0.05) followed by False Discovery Rate controlling procedure of Benjamini and Hochberg (*q *< 0.05). Among them, 23 are three-copy genes, indicated in bold.

**Table 7:** Additional list of three-copy genes that are over-expressed in the external granule cell layer of Ts1Cje as compared to euploid at P0 (*q *> 0.05)

SYMBOL	Ratio	*p*-value	*q*-value	Chr	GO biological process category
Girk2	1.58	0,000	0,109	16	potassium ion transport
Son	1.48	0,001	0,206	16	
Cryzl1	1.4	0,000	0,084	16	zinc ion binding
Gart	1.37	0,000	0,080	16	purine nucleotide biosynthetic process
Olig1	1.37	0,048	0,996	16	neuron fate commitment
Donson	1.34	0,000	0,109	16	multicellular organismal development
Dscr3	1.33	0,001	0,238	16	vacuolar transport
Olig2	1.3	0,002	0,343	16	neuron fate commitment
Hmgn1	1.28	0,000	0,109	16	establishment and/or maintenance of chromatin architecture
Cbr3	1.25	0,030	0,996	16	metabolic process
Prdm15	1.23	0,002	0,331	16	
Ifnar2	1.16	0,019	0,996	16	cell proliferation

Twelve three-copy genes were found significantly differentially expressed by Student *t*-test (*p *< 0.05) but failed the False Discovery Rate controlling procedure of Benjamini and Hochberg (*q *> 0.05).

### A partial monosomy of MMU12 is present in Ts1Cje mice

**Figure 6:**
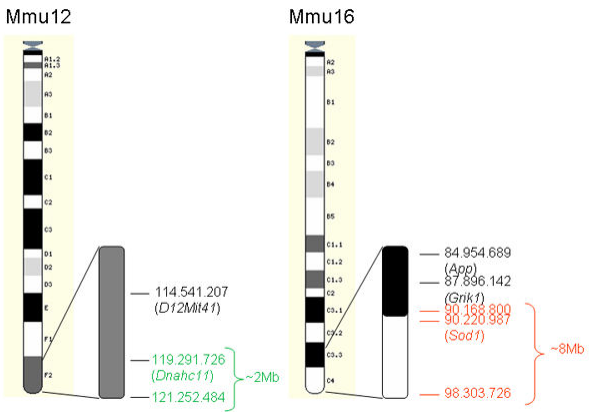
**Partial Mmu16 trisomy and Mmu12 monosomy in Ts1Cje mice**. Representation of the chromosomal breakpoint on Mmu16 and Mmu12 as determined by comparative genomic hybridization using microarrays. Chromosomal locations were deduced by mapping the genomic probes from the microarray on the Ensembl database. Chromosomal locations in green correspond to genes from Mmu12 that have been deleted, in red to the three-copy genes on Mmu16 and in black to euploid genes.

Among the 5 genes whose expression was down-regulated in the external granule cell layer of Ts1Cje mice, 2 were located on the telomeric part of Mmu12: *Cdca7l *and *Sp4 *(Table 6). Their expression ratios (Ts1Cje/euploid) were 0.62 and 0.46 respectively. Since Ts1Cje mice are the result of the translocation of the distal part of Mmu16 from *Sod1 *to *Znf295 *to the telomeric part of Mmu12, we suspected a deletion of these two down-regulated genes. To confirm this chromosomal rearrangement, a comparative genomic hybridization using high resolution (6,4 kb) Agilent genomic microarrays was performed. A 50% reduction of the genomic content corresponding to the chromosomal segment on Mmu12 located between positions 119 291 726 and 121 252 484 was found (Ensembl release 48-Dec 2007 [4]). The deleted part represented about 2 Mb and contained 5 genes: *Dnahc11*, *Sp4*, *Sp8*, *Abcb5 *and *Itgb8*. All of them are related to either neuronal proliferation or maturation, embryogenesis or peripheral and central nervous system development. The additional translocated MMU16 segment started at position 90 168 800, just proximal to the *Sod1 *gene (90 220 987) and spanned to position 98 303 726 at the telomeric end of Mmu16. This genomic interval represented about 8 Mb (Figure 6 and see Additional file 5).

## Discussion

DS is caused by an aneuploidy corresponding to the trisomy or partial trisomy of chromosome 21. The publication of the DNA sequence of the human genome revealed the presence of about 350 genes on chromosome 21. Although this number is low as compared to other human autosomes, it is still too large to develop transgenic mouse models for all of them. Moreover transgenic mouse models for single genes are not adequate to address fundamental questions on gene interactions. Thus in order to find the genes that contribute to the DS phenotype and to the phenotypic variability, gene expression profiling has been applied to mouse models with segmental duplications of DNA segments orthologous to human chromosome 21. The studies published so far were conducted in different mouse models, on different tissues and at various developmental stages. In addition the technology and the statistical tools were different thereby reducing the possibility of grouping the analysis and making insights into the genotype-phenotype status of DS. We thus designed a gene expression study in which we could measure the effects of trisomy 21 in the Ts1Cje mouse model, on a large number of samples, in a tissue that is affected in DS where we could quantify the effetcs during postnatal development in order to correlate gene expression changes to the phenotype observed.

DS affects many aspects of brain development and represents the most common cause of inherited human mental retardation. Motor deficits are among the most frequently occurring features of DS. Individuals with DS exhibit hypotonia and motor dysfunction that could be related to cerebellar dysfunction [33,34]. DS patients present a consistent reduction of the total brain volume with a disproportionally greater reduction in the cerebellum [35,36]. One of the major findings in Ts65Dn and Ts1Cje mouse models of DS carrying segmental duplications, is the significant reduction in cerebellar volume paralleling that observed in DS patients [18,19]. This cerebellar hypoplasia likely results from a decrease in the density of granule cells, the most abundant neuronal cell population. Moreover, behavioural studies have shown motor dysfunctions in Ts65Dn that are more severely affected in tasks involving a high degree of balance and coordination [37]. This hypoplasia occurs during cerebellar postnatal development in the first weeks after birth and does not result from a neurodegenerative process [19,20]. More recently, a reduction of about 20% in the number of granule cell precursors has been reported at birth (P0) and 6 days after birth (P6) in the cerebellar external granule cell layer of Ts65Dn mice [20]. This reduction is much more significant at P0 (*p *= 0.001) than at P6 (*p *= 0.04). In the present study we have shown a 17% significant decrease of volume in the cerebellum of adult Ts1Cje mice and a 32.6% significant decrease of granule cell precursor proliferation only at birth and not at P3 or P7. We analyzed global gene expression during postnatal development of the cerebellum in Ts1Cje mice at the time when major developmental changes occur: at birth (P0) at P3, P7 and P10. During this period granule cells proliferate, migrate from the external to the internal layer of the cerebellum, and differentiate. Our previous study performed at P0, P15 and P30 showed a clear over expression of triplicated genes in the cerebellum of Ts1Cje mice and a small effect on the expression of euploid genes [8,10]. However since the proliferative external granule cell layer disappears by P15 [38], this first study was not designed to detect gene expression changes between P0 and P15. To increase the statistical power of gene expression analysis, we used a large number of samples: 56 cerebella from Ts1Cje and euploid mice bred on the mixed (B6C3SnF1/Orl) genetic background. We performed an ANOVA with a type I error risk of 5% and we found between 2.4% to 7.5% of genes regulated at each time point including some three-copy genes. Then we applied a false discovery rate (FDR) with a 0.05 threshold (5%) on the gene list and found no significant gene at all time points, not even three-copy genes. By increasing the FDR threshold up to 0.40 we found only 47 significant genes among which 6 are three-copy genes (Additional file 3, genes in grey boxes). These results suggest a limited secondary effect of trisomy on euploid genes.

Most three-copy genes were not found significantly differentially expressed suggesting that compensatory mechanisms take place during postnatal development. Only 6 three-copy genes were constantly over-expressed in the cerebellum of Ts1Cje mice at P0, P3, P7 and P10. Using qPCR, we confirmed the increased expression of six three-copy genes at P0. Four of these genes (*Atp50*, *Ifngr2*, *Sod1 *and *Son*) were also found over-expressed in the study of Mao et al. conducted on DS foetal tissues, which included three cerebella [9].

We then performed a second transcriptome analysis using dissected cerebellar regions enriched in granule cell precursors from 9 Ts1Cje and nine euploid mice at P0, at the time when we observed a significant decrease of cell proliferation. About 4% of all genes were significantly differentially expressed in Ts1Cje samples as compared to euploid samples. This value was close the ones obtained in the entire cerebellum at P0, P3, P7 or P10. All the expressed three-copy genes (35) were significantly over-expressed in Ts1Cje with 12genes showing expression ratio significantly lower than 1.5 (*t*-test, *α *= 5%, data not shown). After multi-test correction using the FDR procedure, 32 genes were still significantly modulated in Ts1Cje mice. Among them 23 are three-copy genes, indicating a major primary effect of trisomy on the three-copy genes.

From the euploid genes, 2 map to a chromosomal deletion (see below) and are under-expressed. The other seven are not well characterized and could be new gene candidates for cerebellar DS phenotypes and cerebellum postnatal development. By using an enriched and more homogeneous cell population (the granule cell precursors), we hoped to clarify the secondary effects of trisomy. Surprisingly, not only did we observed a main primary gene dosage effect (23 three-copy genes genes out of 32) but we also identified for the first time a deletion in the Ts1Cje mice (see below). Alternatively it is possible that the secondary effect of trisomy is variable among cells or since we demonstrated a proliferation defect in the granule cell precursors, it is plausible that we failed to identify cell cycle-regulated genes because cells from tissue are not synchronized.

In addition to the three-copy genes, we were able to detect a gene dosage effect on two genes mapping to mouse chromosome 12 (Mmu12), close to the translocation breakpoint of the trisomic segment. A 2 Mb segment of Mmu12 was found to be deleted in Ts1Cje mice. This segment contains 5 genes: *Dnahc11*, *Sp4*, *Sp8*, *Abcb5 *and *Itgb8*. *Sp4 *is a member of a family of zinc finger transcription factors and is highly expressed in the developing hippocampus and cerebellum, in particular in granule cells [39]. Knock-down of *Sp4 *leads to an increased number of highly branched dendrites during maturation of granule cells in the cerebellar cortex [39]. A reduced cell proliferation in hippocampus but not cerebellum has also been reported in *Sp4 *null mutant newborns [40]. Thus, *Sp4*, the only gene found expressed in the external granule cell layer is more likely to affect neuron maturation than proliferation. However it is unlikely that the cerebellum phenotype observed in Ts1Cje mice results from the Mmu12 deletion since a very similar phenotype is present in the Ts65Dn mouse model that do not show any rearrangement on Mmu12. We found a decrease in *Cdca7l *gene expression, a transcription factor involved in cell apoptosis [41] and medulloblastoma transformation [41]. Since *Cdca7l *is located approximately 200 kb proximal to the translocation breakpoint on MMU12 (Figure 6) we concluded that the gene dosage effect extends to regions that are close to the chromosomal rearrangement.

We then looked for genes that were regulated during normal postnatal development of the cerebellum and that were also differentially expressed between Ts1Cje and euploid cerebellum at one of the postnatal stages tested. Among the 1187 genes found, only three were triplicated: *Olig1*, *Dscam *and *Girk2*. *Olig1 *encodes a basic helix-loop-helix transcription factor that is expressed in both the developing and mature vertebrate central nervous system. *Olig1 *has critical function during the formation of motor neurons and oligodendrocytes of the ventral neural tube (review in [24]). *Dscam *encodes an axon guidance molecule that is expressed by neurons in the central nervous system during development and throughout adult life. Its expression in the developing cerebellum is stronger in Purkinje neurons. A *Dscam *mutation in mice leads to a subtle defect in the caudal folium of the cerebellum [42]. *Girk2 *is the only gene over-expressed in Ts1Cje across postnatal development of the cerebellum. It is also over-expressed in the external granule cell layer of Ts1Cje newborns. *Girk2 *is a member of the G protein-gated inwardly rectifying potassium channel family that regulates cellular excitability and neurotransmission. It is mainly expressed in the cerebellum granule cells [22]. A missense mutation in *Girk2 *leads to abnormalities of the cell cycle and apoptosis in the external granule cell layer of the cerebellum in the weaver mice [43]. The *Girk2 *mutated gene encodes channels that exhibit loss of potassium selectivity. In contrast, *Girk2 *knock-out mice are morphologically indistinguishable from wild-type mice, suggesting that the weaver phenotype is likely due to abnormal *Girk2 *function [44]. Ion channels can regulate cell cycle and implication of transmembrane potassium fluxes via inward rectifier channels in the regulation of cell cycle has been proposed [45].

Moreover it has been hypothesized that over-expression of the *Girk2 *subunit in trisomic mice will likely produce a hyperpolarization of the membrane [46]. Depolarization enhances calcium entry via voltage-sensitive Ca^2+ ^channels and activates calmodulin kinase and calcineurin phophatase. The activation of calcineurin induces many genes encoding extracellular and intracellular signalling molecules involved in granule cell development. [47]. Thus, hyperpolarization of the membrane may prevent or reduce calcium entry in cells and decrease cell proliferation.

To further identify gene candidates for the DS cerebellar phenotype, we selected gene candidates involved in genetic regulation of the proliferation of the granule cell precursors [26] and looked at their expression in the cerebellum of Ts1Cje and euploid mice at birth. Sonic hedgehog (Shh) is secreted by Purkinje cells and regulates proliferation of the granule cell progenitors [38]. The mitotic response of cultured granule cell precursors to Shh was shown to be reduced in Ts65Dn mice as compared to euploids [20]. Systemic treatment of newborn Ts65Dn mice with an agonist of the Sonic Hedgehog pathway increased the mitotic index of trisomic granule cell precursors and even restored the granule cell population in one week.

However, to date, genes involved in the Shh pathway have not been assigned to human chromosome 21 nor have they been shown to be differentially expressed in trisomic mice or in people with DS. Thus the positive effect of treatment with agonists of the Shh pathway could result from an over-stimulation of Shh receptors that are present on granule cell precursors. It remains to be shown whether this positive effect will normalize the cerebellar volume and improve the cerebellar-related behavioural deficits observed in Ts65Dn mice [37].

Other genes have been reported to regulate the proliferation of granule cell progenitors. Some interact with the Shh pathway, such as *Numb *which is a suppressor of Shh signaling [28]. *Numb *also regulates Notch1 pathway [28,48]. Notch signalling is another crucial development-regulated pathway that appears to maintain cells in an undifferentiated state in the early mammalian central nervous system [49]. In addition, we tested other genes related to granule cell proliferation or neural proliferation including *IGF-I *[50], *bFGF *[51], *Pax6 *[52], *Olig1 *and *Olig2 *[52]. We were not able to show any consistent difference of expression for any of these genes except a slight but significant over-expression of *Shh*. Functional profiling of the differentially expressed genes from microarray experiments didn't reveal any enrichment in gene ontology categories related to cell proliferation.

Two genes over-expressed in the external granule cell layer of Ts1Cje mice at birth, *S100a6 *and *Dlk1*, encode calcium ion binding proteins. *S100a6 *belongs to the S100 family proteins that play an important role in cell growth, differentiation, and motility through calcium-dependent signalling pathways [53]. *S100a6 *encodes the calcyclin protein whose expression is associated with neuronal differentiation [54,55]. This gene is thus a candidate for the cerebellar hypoplasia. *Dlk1 *(delta-like) is a transmembrane and secreted protein from the epidermal growth factor-like homeotic family. *Dlk1 *expression is increased in gliomas and over-expression in transfected cells promotes cell proliferation by inducing the expression of cyclin D1, CDK2, and E2F4 [56]. However, we could not detect any change in expression of any of these genes suggesting that either *Dlk1 *has no effect on granule cell proliferation or that the effect is not visible because of lack of cell synchronization in tissues.

Recently, the generation of a collection of haploid yeast strains that each bear an extra copy of one or more of almost all of the yeast chromosomes has been published [57]. Their characterization revealed that aneuploid strains share a number of phenotypes, including defects in cell cycle progression, that are independent of the identity of the individual extra chromosomes. It was thus proposed that disruption of cell homeostasy in DS could be due to the additional chromosomal material rather than the gene content [58]. However aneuploidy is a condition frequently found in tumor cells which often display high rate of proliferation, suggesting that yeast and mammalian cells can respond differently to aneuploidy. Moreover in DS, reduced proliferation rates have not been observed in all proliferative cells across development and in adulthood. We show here a reduced proliferation of granule cell precursors only at birth.

Several studies have reported evidence of neurogenesis impairment in the developing neocortex, in the dentate gyrus of DS foetuses and in mouse models [59-62]. In the dentate gyrus, cell proliferation is decreased in aged but not young adult Ts65Dn mice [62] suggesting a neurodegenerative related process occurring in trisomic mice. Another study has reported a lower cell proliferation due to cell cycle alteration in the dentate gyrus of foetuses with DS and in mouse models. Using different markers of proliferation the authors showed that both DS foetuses and P2 Ts65Dn mice have a higher number of proliferating cells in G2 and a smaller number of cells in the M phase of the cell cycle [59]. A similar result was reported in the forebrain of Ts65Dn embryos [60] where delayed expansion of neocortical layers and reduced growth of the hippocampus were found to be correlated with a slower cell cycle. Interestingly, their results suggest that the cell cycle abnormality, occurring in the trisomic ventricular zone of the neocortex but not in the hippocampus, might be developmentally regulated or compensated during neurogenesis. Thus, neurogenesis impairment occurs in different areas of the developing brain in trisomy 21 but not continuously, suggesting that genes involved might be different depending on the brain structure and the developmental stage.

## Conclusion

High throughput gene expression analysis in the cerebellum of a large number of Ts1Cje and euploid mice demonstrates a prevailing gene dosage effect of trisomy and a limited secondary effect across postnatal development. Moreover by using an enriched cell population involved in cerebellar hypoplasia in DS we were not able to detect a global change in gene expression. 80% of gene expression differences were attributed to dosage imbalance, suggesting that the three-copy genes are likely to be directly responsible for the phenotype present in cerebellum.

By correlating the effects of trisomy and those of postnatal development on gene expression, we identified only 6 three-copy genes that are constantly over-expressed in Ts1Cje mice during early postnatal development of the cerebellum, and only 3 three-copy genes that are regulated during normal postnatal development of the cerebellum and are over-expressed in the Ts1Cje mice. Only *Girk2 *is common to both of these groups suggesting that this inwardly rectifying potassium channel expressed in granule cells in the cerebellum could be involved in cerebellar hypoplasia in DS. We propose that reducing the expression of *Girk2 *in the Ts1Cje mice could help to recover a normal cerebellum phenotype.

## Methods

### Mice

Ts1Cje male mice carrying a segmental duplication of the MMU16 region from *Sod1 *to *Znf295 *were bred with wild-type females on a B6C3SnF1/Orl mixed background. Ts1Cje mice were genotyped using polymerase chain reaction (PCR) primers for neomycin (*Neo*), forward: TATTCGCTATGACTGGGCACAAC and reverse: TTCAGTGACAACGTCGAGCACA. Day of plug is reported as E0 and day of birth as P0.

### In Vivo magnetic Resonance Imaging

Six male Ts1Cje adult mice and nine male euploid from 4 litters were anaesthetised (2% isofluorane) and imaged with a 7 Tesla MRI animal scanner (Bruker Pharmascan, Ettlingen, Germany). A TurboRare-3D sequence was used with the following parameters: field of view of 2 × 2 × 2 cm with a matrix of 196 × 196 × 196 that results in an isotropic resolution of 102 *μ*m, TR/TE = 1500/43 ms, 2 averages. Total acquisition time was 1h58. Respiration was monitored during the acquisition. Brain and cerebellum volumes were calculated using the freeware InsightSNAP . The obtained segmentation using implemented snake evolution algorithm was manually corrected in the three orthogonal planes for all cases.

### Histological Analysis

After intracardiac perfusion of mice with paraformaldehyde 4% in 0.1 M PBS, brains were removed and cryoprotected in 30% sucrose for 2 days and then stored at -80°C. Frozen brains were cut in the parasagittal plane (16 *μ*m-thick sections) on a cryostat (MicromMicrotech HM550). Immunostaining was performed using the cell-cycle-associated protein antibody rabbit anti-Ki67 (NovoCastra, ref. 2010-09) [21] and secondary antibody biotinylated anti-rabbit IgG (Valbiotech). Sections were blocked for 1 h in PBS containing 0.2% gelatin [G], 0.25% Triton X-100 [T], 0.1% sodium azide [A] and 0.1 M lysine. The sections were incubated overnight at RT with the primary antibody at 1:1000 in PBS-GTA. After washes in PBS-T, the second antibody was incubated at 1:200 in PBS-GT for 2 h at RT. For revelation, Streptavidin Biotinylated Horseradish Peroxydase Complex (Amersham Pharmacia Biotech) was prepared at 1:400 in PBS-G and incubated for 2 hours at RT followed by 30 mg/ml DiAminoBenzidine (DAB) in Tris 0,1 M pH 7.6 + H2O2 0.05% during about 12 min. Counterstaining was made by Cresyl Violet coloration (Cresyl-violet 1% + thionine 1% solution). After dehydratation in graded ethanol and Xylene, slides were mounted in Eukitt mounting medium (Flucka Biochemika). Photomicrographs were taken on a Leica microscope equipped with a QICam Fast 1934 CCD camera (QImaging). Cells labelled with DAB (dark brown) and cresyl (blue) were counted separately with Image-Pro Plus software. The index of proliferation was defined as the ratio between Ki67^+ ^cells on cresyl-violet^+ ^cells.

### Cerebellum RNA labelling and microarray hybridization

Total RNA was extracted from frozen individual cerebella and treated with DNAse using RNeasy Minikit (Qiagen) in accordance to the manufacturer's protocol. The quality and quantity of each RNA sample was checked using the Agilent 2100 Bioanalyzer with RNA 6000 NanoChips (Agilent Technologies). Five *μ*g of total RNA from each cerebellar sample were converted to cDNA (Verso cDNA kit, ThermoScientific) in the presence of amino allyl-dUTP, purified on Qiaquick columns in accordance with the manufacturer's protocol (Qiagen), incubated for 10 min in presence of NaHCO3 0.05 M (Sigma) and 1 h with NHS-ester Cy3 or Cy5 (GE Healthcare). After purification, hybridization of labelled sample pairs on RNG/MRC mouse pangenomic microarrays (25 K, [63]) was performed in hybridization buffer (50% formamide, 4× saline sodium citrate [SSC], 0.1% SDS, and 5× Denhart) at 42°C overnight. Slides were washed in 2× SSC and 0.1% SDS for 5', 1× SSC for 5', 0.2× SSC for 5', 0.05× SSC for 5' and then dryed by centrifugation 4' at 800 rpm. Data were acquired on the 2-laser Scan Array Gx (Perkin Elmer) with a resolution of 5 *μ*m and analyzed with GenePix Pro 4.1 software (Axon). For each array, the raw data comprised the median feature pixel intensity at wavelengths 635 nm and 532 nm for Cy5 and Cy3 labelling, respectively. After subtraction of the background signal, local LOWESS normalization of the M values corresponding to Cy5/Cy3 signal ratios in log2 was applied under the R freeware . EXPERIMENTAL DESIGN: cDNA from 28 Ts1Cje and 28 euploid mice at P0 (6), P3 (8), P7 (8) and P10 (6) were hybridized on 28 microarrays with the following conditions: on the same microarray we always compared a Ts1Cje sample versus an euploid sample and samples from mice of the same age or with a maximum difference of 4 days (P0 versus P3, P3 versus P7 or P7 versus P10) (see Additional file 2). All the microarray data have been deposited on the GEO database under the accession number GSE11448.

### External granule cell layer RNA labelling and microarray hybridization

Total RNA from frozen individually dissected external granule cell layers was extracted and checked as above. Fifty ng of RNA were converted to cDNA then to biotinylated cRNA, labelled with Cy3 and hybridized to Mouse-6 Expression BeadChips (Illumina) on the Integragen Illumina microarray platform (Evry, France), according to the Illumina procedures. Scans were performed on the BeadStation 500 scanner (Illumina) with a 0.8 *μ*m resolution at a wavelength of 532 nm and data were extracted with the Beadstudio software (Illumina). Raw data, corresponding to the mean signal intensity of up to 60 spots have been normalized by the mean signals of the 18 microarrays. This experiment included 9 Ts1Cje and 9 euploid mice at P0 from three litters and cRNAs from each litter were hybridized on a slide of 6 arrays. All the microarray data have been deposited on the GEO database under the accession number GSE11472.

### Statistical analysis

All manipulations and statistical analysis were implemented with the R freeware. FILTERING: for the RNG/MRC microarray results, all the control spots, irregular spots and spots with signal intensity less than 1.2 fold the local background were excluded. For Illumina beadchip data, all the spots with a detection *p*-value higher than 0.01% were excluded. ANALYSIS OF VARIANCE (ANOVA): to list differentially expressed genes across normal cerebellar development and between trisomic and euploid mice at each step of development, we performed an ANOVA with 2 factors: development and genotype (*α*i and *β*i parameters respectively in Table 2). We deduced the *p*-values for the two parameters from comparison with 0 under the null hypothesis. To find differentially expressed genes between Ts1Cje external granule cell layer at P0 and controls, we performed Student's *t*-tests. *p*-values were deduced from comparison with 0 under the null hypothesis and corrected using the False Discovery Rate (FDR) controlling procedure of Benjamini and Hochberg:  for i = m-1 to 1, where m is the number of tests and i the rank of the ascending ordered *p*-values. PRINCIPAL COMPONENT ANALYSIS (PCA): Samples from Illumina arrays were defined by the normalized signal intensities of the spots. PCA was processed using either all expressed genes or only the three-copy genes. ENRICHMENT: significant enrichments and/or depletions of GO categories among genes of interest were obtained using the web-based tool eGOn (explore GeneOntology, ) with a *p*-value of 5%. Only GO categories with at least 3 genes were considered.

### Real-time quantitative PCR

To validate expression ratios obtained from microarray analysis, 1 *μ*g of total RNA from 6 euploid and 6 trisomic cerebellar samples at P0 were individually converted into cDNAs overnight at 37°C (Verso cDNA kit, ThermoScientific). Quantitative PCR on 7 Mmu16 genes (*Dscam*, *Son*, *Olig1*, *Dyrk1a*, *Girk2*, *Rcan1*, *Hmgn1*) and 17 genes mapping to other chromosomes (*Nfat1*, *Nfat5*, *NR1*, *Cd24a*, *Shh*, *Ptch1*, *Gli1*, *Gli2*, *Ift88*, *Numb*, *Itch*, *Npc1*, *Notch2*, *Ccna2*, *Psen2*, *Itga6*, *Pax2*, *Actb*) were performed as Taqman assays in an LightCycler 480 System (Roche Molecular Biochemicals) in the presence of 0.4 *μ*M of each specific primer (designed by Roche ProbeFinder software), 0.2 *μ*M of probe (Roche Universal Probe Library Set), 1× LightCycler TaqMan Master mix containing 3.5 mM Mg^2+^, 200 *μ*M dNTP mix and Hotstart Taq polymerase. Results were normalized using *Actb *mapping to Mmu5 as a reference gene. Primer sequence and probe number (UPL) are available in Additional file 6.

### Comparative Genomic Hybridization experiments

Array-CGH was performed using the Agilent Mouse Genome CGH Microarray Kit 244 k (Agilent Technologies). This microarray contains about 235 000 probes that allow genome-wide copy number variation profiling with an average resolution of 6,4 kb. Labelling, hybridization and analysis were performed by DiagnoGene (France) following the protocols provided by Agilent. Briefly, 1.5 *μ*g of purified DNA of 2 Ts1Cje and 2 euploid mice were double-digested with *Rsa*I and *Alu*I. Each digested sample was labelled by random priming using Cy5 for the trisomic samples and Cy3 for the controls. Hybridizations were performed at 65°C for 40 h. The arrays were analyzed with the Agilent scanner and the Feature Extraction software. Genomic rearrangements were obtained using the CGH Analytics software.

## Authors' contributions

JL carried out the molecular genetic studies. JL, BL and RS carried out the MRI studies. FP, RW and ID carried out the immunoassays. TV and RW carried out the EGL dissections. LD participated in the molecular genetic studies. RXM participated in the immunohistochemical studies. JL, IR and MCP participated in the design of the study and performed the statistical analysis. JL, MCP and JR drafted the manuscript. All authors read and approved the final manuscript.

## Supplementary Material

Additional File 1 Using magnetic resonance imaging we measured the volumes of whole brain and cerebellum of six male adult Ts1Cje and nine euploid littermates with a very high-resolution of 100 *μ*m. The obtained segmentation (C) using implemented snake evolution algorithm was manually corrected in the three orthogonal planes: horizontal (A), sagital (B) and coronal (D). Cb: cerebellum. Click here for file

Additional File 2 Fifty six samples from individual cerebella at P0, P3, P7 and P10 were hybridized on 28 two-color microarrays. On each microarray we hybridized a Ts1Cje sample versus an euploid sample. In addition on the same microarray we compared samples from mice of the same age or with a maximum difference of 4 days (P0 versus P3, P3 versus P7 or P7 versus P10). Each sample is identified by a letter and a number referring to the litter and to the pup in the litter respectively. Trisomic and euploid samples at each stage of development were equally labelled by Cy3 (in green) and Cy5 (in red). Click here for file

Additional File 3 For each gene, the expression ratio, the *p*-value of the ANOVA (*α *= 5%) or the Student *t*-test (*α *= 5%) and the gene ontology are given. In bold are three-copy genes and in grey are genes that passed the False Discovery Rate controlling procedure of Benjamini and Hochberg (*q *< 0.40). Click here for file

Additional File 4 For each gene, we indicate the Ts1Cje/euploid ratio at P0, P3, P7 and P10 and the expression ratio between P0–P3, P0–P7 and P0–P7 in euploid mice. In grey, non significant results (*p *> 0.05). NE: non expressed. Click here for file

Additional File 5 In green are genes significantly downregulated and in red genes significantly overexpressed (Student *t*-test, *α *= 5%). NE: non expressed. Genes are ranked according to their chromosomal location (top to bottom: centromeric to telomeric). Click here for file

Additional File 6 UPL refers to the number of the probe in the Roche Universal Probe Library. Click here for file
