# Supplementary figures and images for: Gene expression signature of cerebellar hypoplasia in a mouse model of Down syndrome during postnatal development
Source: BMC Genomics. 2009 Mar 30;10:138. doi: 10.1186/1471-2164-10-138 (PMC2678156; doi:10.1186/1471-2164-10-138)

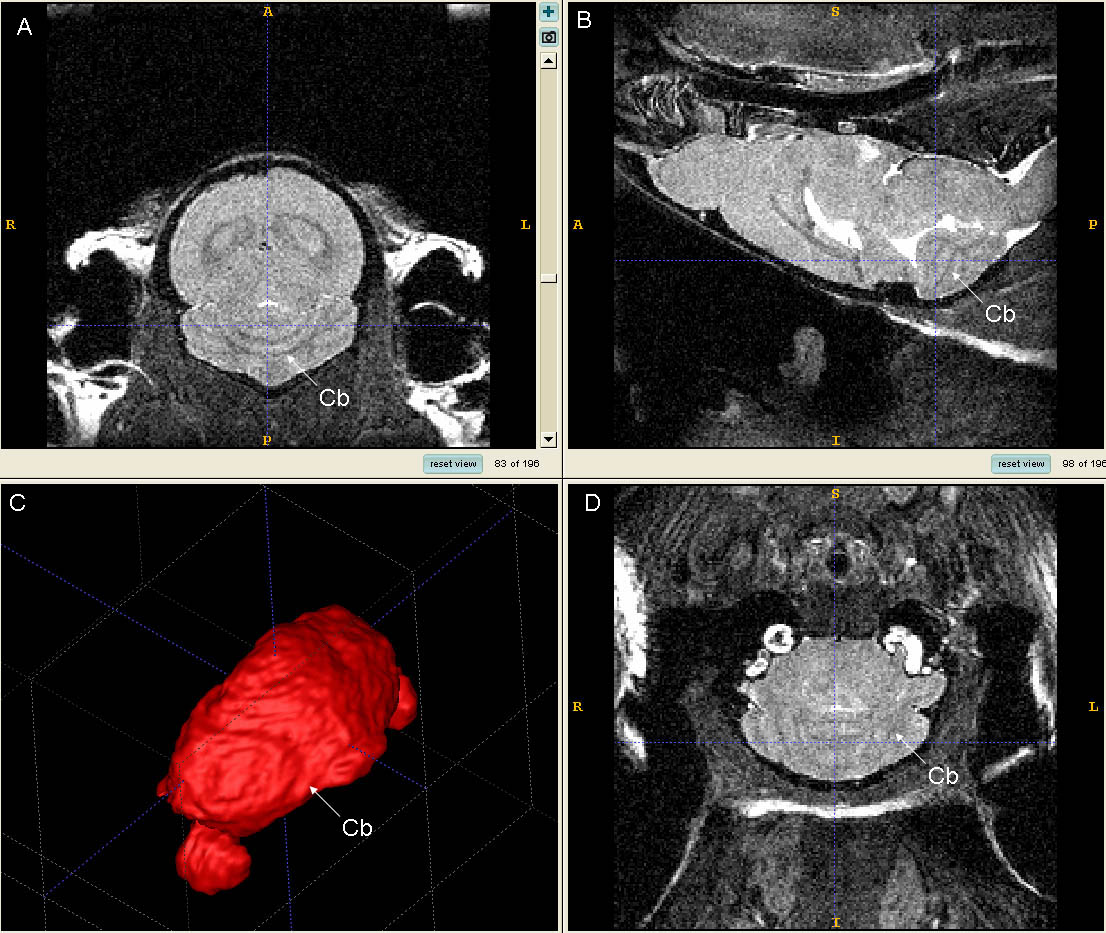

Supplement: Additional File 1 — Using magnetic resonance imaging we measured the volumes of whole brain and cerebellum of six male adult Ts1Cje and nine euploid littermates with a very high-resolution of 100 μm. The obtained segmentation (C) using implemented snake evolution algorithm was manually corrected in the three orthogonal planes: horizontal (A), sagital (B) and coronal (D). Cb: cerebellum. [file 1471-2164-10-138-S1.jpeg]
